# Proportions, trends, and outcomes of posterior circulation ischemic stroke in the United States

**DOI:** 10.3389/fneur.2026.1776618

**Published:** 2026-05-13

**Authors:** Adnan I. Qureshi, Nived Jayaraj Ranjini, David Cohen, Shahhan Spall, Akash Roy, Christy N. Cassarly, Renee H. Martin, William J. Powers, Chun Shing Kwok

**Affiliations:** 1 Zeenat Qureshi Stroke Institute, Columbia, MO, United States; 2 Department of Neurology, University of Missouri, Columbia, MO, United States; 3 Department of Public Health Sciences, College of Medicine, Medical University of South Carolina, Charleston, SC, United States; 4 Department of Neurology, Duke University School of Medicine, Durham, NC, United States; 5 Mid Cheshire Hospitals NHS Foundation Trust, Leighton Hospital, Crewe, United Kingdom

**Keywords:** anterior circulation ischemic stroke, national survey, posterior circulation ischemic stroke, thrombectomy, thrombolysis

## Abstract

**Background:**

Posterior circulation ischemic stroke has been previously studied in single-center and multicenter registries. The contemporary proportions, trends, and outcomes of posterior circulation ischemic stroke at a national level are not known.

**Methods:**

We identified patients admitted with ischemic stroke using the Nationwide Inpatient Sample from 2016 to 2022. We compared the rates of intravenous (IV) thrombolysis, thrombectomy, in-hospital mortality, routine discharge without palliative care (based on discharge disposition), and length and costs of hospitalization in patients admitted with posterior circulation ischemic stroke to patients admitted with anterior circulation ischemic stroke.

**Results:**

Among 4,773,715 ischemic stroke admissions, 1,675,030 (35.1%) had a territory-specific localization code and 1,630,520 were included for analysis. A total of 383,990 (23.6%) and 1,246,530 (76.4%) patients were admitted with posterior and anterior circulation ischemic stroke, respectively. From 2016 to 2022, the number of patients admitted with posterior circulation ischemic strokes increased from 42,055 in 2016 to 73,755 in 2022, demonstrating a temporal trend (p trend *p* < 0.001). Patients with posterior circulation ischemic stroke were more likely to have diabetes mellitus and less likely to have heart failure and atrial fibrillation. The proportion of patients with a National Institutes of Health Stroke Scale (NIHSS) score of 0–4 was significantly higher in patients with posterior circulation ischemic stroke (63.8% vs. 40.8%). The utilization of IV thrombolysis (6.1% vs. 13.3%) and thrombectomy (4.0% vs. 16.4%) was lower in patients with posterior circulation ischemic stroke compared with those with anterior circulation ischemic stroke. The in-hospital mortality was significantly higher [odds ratio 1.53, 95% CI: (1.45–1.63), *p* < 0.001] in patients with posterior circulation ischemic stroke after adjusting for potential confounders, including NIHSS score strata.

**Conclusions:**

Over 20% of acute ischemic strokes occur in posterior circulation territories in the United States. Patients with posterior circulation ischemic stroke are less likely to receive acute stroke treatment compared with those with anterior circulation ischemic stroke.

## Introduction

The 2025 Heart Disease and Stroke Statistics report that approximately 795,000 people experience a new or recurrent stroke every year in the United State of America (USA), of which 87% or 691,650 are ischemic strokes (1). About 20–25 percent of all ischemic strokes are estimated to be posterior circulation ischemic strokes (2–5). Most of the existing data evaluating posterior circulation ischemic strokes is based on single-center studies (4) or multicenter registries (6, 7). Studies have suggested a high burden of cardiovascular risk factors, such as diabetes mellitus (6, 7) and high early risk of recurrence in patients with posterior circulation ischemic stroke, particularly in those with intra- or extra-cranial stenosis (8, 9). There has been renewed interest in posterior circulation ischemic stroke due to the availability of intravenous thrombolysis (10), thrombectomy (11), and stent placement (12). Extracting and publishing the estimates is essential to raise public awareness, address disparities, identify research gaps, allocate resources effectively, and develop preventive and treatment strategies. This is the first contemporary national analysis to provide estimates on the national trends in occurrence, and short-term outcomes in patients with posterior circulation ischemic stroke, incorporating treatment utilization and National Institutes of Health Stroke Scale (NIHSS) score stratified outcomes.

## Methods

The National Inpatient Sample (NIS), developed by the Healthcare Cost and Utilization Project (HCUP) under the Agency for Healthcare Research and Quality, is not a census of U.S. residents but a 20% stratified probability sample of discharges from U.S. community hospitals (13). When survey discharge weights are applied, the NIS is designed to approximate inpatient hospitalizations from hospitals serving more than 97% of the U.S. population. Importantly, the NIS sampling frame and weighting methodology remained structurally stable between 2016 and 2022 without any major redesign that would materially affect national representativeness. Although total hospitalizations declined in 2020 due to the Coronavirus Disease 2019 pandemic, this reflected a true reduction in healthcare utilization rather than a change in sampling strategy or coverage. Therefore, the proportion of the USA population represented by the NIS hospital sampling remained stable from 2016 through 2022, and temporal trends observed in this analysis are unlikely to be attributable to changes in database design or representativeness (14–16). We prepared this manuscript in accordance with the recommendations of the Strengthening the Reporting of Observational Studies in Epidemiology (STROBE) statement (17).

### Patient identification

We identified patients with posterior circulation ischemic stroke using the following International Classification of Diseases (ICD)-−10 diagnosis codes: I63.432, I63.11, I63.12, I63.213, I63.541, I63.011, I63.012, I63.013, I63.019, I63.01, I63.02, I63.34, I63.542, I63.531, I63.442, I63.549, I63.441, I63.543, I63.431, I63.432, I63.433, I63.532, I63.21, I63.22, I63.533, I63.219, I63.211, I63.443, I63.212, I63.332, and I63.331. We identified patients with anterior circulation ischemic stroke using the following ICD-10-diagnosis codes: I63.512, I63.51, I63.513, I63.519, I63.412, I63.411, I63.312, I63.311, I63.319, I63.419, I63.522, I63.521, I63.529, I63.422, I63.421, I63.429, I63.232, I63.231, I63.321, I63.322, I63.329, I63.131, I63.132, I63.133, I63.139, I63.239, I63.03, I63.031, I63.032, I63.033, and I63.039 (see Supplementary Table 1). We excluded hospital admissions for patients aged less than 18 years. Patients with diagnosis codes for both posterior and anterior circulation ischemic stroke were also excluded from the analysis.

### Variables used

A full description of the variables used in the analysis is presented in Supplementary Table 1. We identified patients who underwent thrombectomy and cerebral angiogram using ICD-10-procedure codes (thrombectomy: 03CG3ZZ, 03CG3Z7, 03CG4ZZ, 03CH3Z7, 03CJ0ZZ, 03CJ3ZZ, 03CK3Z7, 03CK3ZZ, 03CL3Z7, 03CL3ZZ, 03CL0ZZ, 03CP3ZZ, 03CY3ZZ, 00C73ZZ; cerebral angiogram: B3181ZZ). We also identified patients who underwent vertebral artery stent placement using the following ICD-10-procedure codes- 037Q34Z, 037Q35Z, 037Q3DZ, 037P3ZZ, 037P34Z, or 037P3DZ (with or without 03CQ3ZZ and 03CP3ZZ), carotid endarterectomy (03CL0ZZ, 03CJ0ZZ, 03CH0ZZ, 03CK022), and carotid stent placement (037L3^*^, 037K3^*^, 037J3^*^, 037H3^*^).

### Validation of codes

We evaluated the diagnostic performance of ICD-10 discharge diagnosis codes for identifying ischemic stroke territory (posterior vs. anterior circulation) in 200 consecutive patients admitted with acute ischemic stroke to the University of Missouri Medical Center (2019–2020). The validation study was conducted in accordance with the ICD-10-CM codes utilized for the NIS sample. By applying the same coding architecture, specifically for posterior and for anterior circulation ischemic strokes, we ensured methodological consistency across both the national database analysis and the clinical validation cohort. Stroke territory was adjudicated by a board-certified vascular neurologist who was blinded to ICD-10 coding at the time of the review. Territory assignment was based on clinical documentation and computed tomography (CT) and/or magnetic resonance imaging (MRI) findings using predefined vascular territory criteria. Posterior circulation ischemic stroke was defined as infarction within vertebrobasilar or posterior cerebral artery (PCA) distributions (including brainstem, cerebellum, occipital lobe, medial temporal lobe, and thalamus consistent with posterior supply), and anterior circulation stroke as infarction within internal carotid, middle cerebral artery, or anterior cerebral artery territories. Lacunar and thalamocapsular infarcts were classified according to anatomical location and vascular supply. In cases of fetal PCA or P1 segment variants, infarcts were categorized by anatomical territory rather than embryologic origin. The University of Missouri Institutional Review Board approved the chart review protocol.

### Outcomes

Palliative care and discharge status. Palliative care was identified using the ICD-10 diagnosis code Z51.5 during the index hospitalization. Discharge disposition was obtained from the HCUP standardized discharge disposition variable (DISPUNIFORM) and combined with the palliative care indicator to create a 3-level primary outcome, discharge status, defined as: (1) routine discharge without palliative care, corresponding to discharge to home/self-care; (2) non-routine discharge without palliative care, corresponding to transfer to a short-term hospital, transfer to another institution, or discharge to home health care; and (3) in-hospital death or any discharge/transfer with palliative care, defined as in-hospital mortality or the presence of Z51.5 with any discharge disposition. Additional discharge-based composites. DISPUNIFORM was also used to construct additional composite discharge endpoints as specified in the Outcomes section. Hospital costs. Hospitalization costs were estimated by converting total hospital charges to costs using HCUP-recommended cost conversion, calculated as total charges multiplied by the HCUP cost-to-charge ratio.

### Statistical analysis

Statistical analysis was performed using Stata 13.0 (College Station, TX, USA). We applied discharge weights as per the recommendations of HCUP to generate national estimates. Descriptive statistics were presented separately for the patients with posterior circulation ischemic stroke and those with anterior circulation ischemic stroke. For continuous variables, the median and interquartile range was presented, and for categorical variables, counts and percentages were presented. Baseline characteristics were mostly compared using standardized mean differences (SMDs) (18), defined as the difference in group means (or proportions) divided by the pooled standard deviation (SD) and *p-values* were used for comparing medians (19, 20). Different formulas were used to find the pooled SD for means and proportions. SMDs are not driven by sample size and therefore provide an effect-size measure of group differences and avoid the limitations of using *p-value* which is highly sensitive to sample size and very large samples can yield extremely small *p-values* even for trivial differences (21). We used the threshold of ≥0.10 to denote a potentially meaningful difference.

The “ptrend” STATA function was used to assess whether there was a significant trend in admissions for patients with posterior and anterior circulation ischemic strokes from 2016 to 2022. In-hospital mortality and routine discharge (excluding palliative care) were analyzed using logistic regression, and linear regression was used to compare length of stay and cost. All models were adjusted for age, sex, race, diabetes mellitus, hypertension, cigarette smoking, hypercholesterolemia, obesity, atrial fibrillation, heart failure, teaching hospital, and rural hospital. In sensitivity analyses, we also adjusted for NIHSS score, categorized into strata (0–4, 5–15, 15–20, 21–42) (14), which was only available for 50.3% of the patients.

## Results

**Figure 1:**
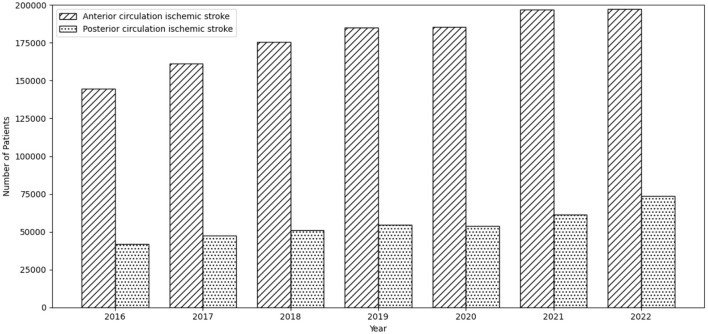
Trends in posterior circulation and anterior circulation ischemic strokes from 2016 to 2022.

Among 4,773,715 ischemic stroke admissions identified in the Nationwide Inpatient Sample from 2016 to 2022, 3,098,685 (64.9%) did not have a territory-specific localization code and were therefore not classifiable as anterior or posterior circulation ischemic stroke. Among the remaining 1,675,030 (35.1%) admissions with a reported localization, 1,630,520 patients were included in the analysis (Supplementary Figure 1). A total of 383,990 (23.6%) and 1,246,530 (76.4%) patients were admitted with posterior circulation and anterior circulation ischemic strokes, respectively, over the 7-year period. There was a progressive increase in the number of patients who were admitted with posterior circulation ischemic stroke from 42,055 in 2016 to 73,755 in 2022, which outpaced anterior ischemic stroke, which increased by 36.5 % in this period (144,640 to 197,440) (Figure 1).

Baseline demographic, clinical, and hospital characteristics were largely comparable between patients with posterior circulation ischemic stroke and those with anterior circulation ischemic stroke (see Table 1). Most variables demonstrated negligible differences, with SMDs below 0.10. Median age showed minimal variation between groups (70 vs. 72 years; *p* < 0.001), and racial distributions were similar (SMDs < 0.05). Hospital-level characteristics, including bed size, geographic region, teaching status, and rural designation, also showed minimal imbalance (SMDs < 0.07). Patterns of admission and socioeconomic indicators, including expected primary payer and ZIP-code–based income quartile (data not shown), were also well balanced (SMDs < 0.08). Cardiovascular risk profiles were highly similar for most comorbidities, including hypertension, hyperlipidemia, cigarette smoking, and chronic kidney disease (SMDs ≤ 0.05). A few variables demonstrated small but meaningful differences. Women comprised a greater proportion of the anterior circulation stroke group (SMD 0.13). Atrial fibrillation was also more prevalent among patients with anterior circulation ischemic strokes (SMD 0.16). Smaller imbalances were observed for diabetes mellitus (SMD 0.08) and heart failure (SMD 0.07). No other comorbidity demonstrated clinically relevant differences.

**Table 1:** Baseline characteristics of patients with posterior and anterior circulation ischemic stroke.

Characteristic^*^	Posterior circulation (*n* = 383,990)	Anterior circulation (*n* = 1,246,530)	SMD
Demographic characteristics			
Age, median (IQR), years	70 (60–79)	72 (61–82)	< 0.001^**†**^
Women, %	44.2	50.7	0.130
Race/Ethnicity, %			
White	68.3	68.8	0.011
African American	15.9	16.7	0.022
Hispanic	9.1	8.0	0.039
Asian/Pacific Islander	3.3	3.2	0.006
Native American	0.5	0.5	0.000
Other	2.8	2.9	0.006
**Hospital characteristics, %**			
Small bed size	15.7	13.4	0.065
Medium bed size	26.3	25.9	0.009
Large bed size	58.0	60.7	0.055
Northeast	18.6	17.8	0.021
Midwest	21.0	21.3	0.007
South	41.3	42.0	0.014
West	19.1	18.9	0.005
Teaching hospital	79.4	81.5	0.053
Rural hospital	16.0	16.6	0.016
**Admission characteristics, %**			
Weekend admission	25.4	26.2	0.018
Elective admission	4.0	4.3	0.015
Transfer admission	19.2	22.4	0.079
**Primary expected payer, %**			
Medicare	61.1	64.8	0.077
Medicaid	10.1	10.1	0.000
Private insurance	21.6	18.6	0.075
Self-pay	4.2	3.7	0.026
**Cardiovascular risk factors, %**			
Cigarette smoking	0.9	0.9	0.000
Alcohol misuse	2.7	2.8	0.006
Hypertension	84.0	84.1	0.003
Hyperlipidemia	54.6	55.0	0.008
Obesity	15.1	14.4	0.020
Diabetes mellitus	39.7	35.8	0.081
Prior myocardial infarction	7.1	7.5	0.015
Heart failure	19.8	22.7	0.071
Atrial fibrillation	26.7	34.0	0.159
Prior stroke	23.2	24.2	0.024
Peripheral vascular disease	3.5	4.2	0.036
Chronic kidney disease	19.4	18.9	0.013
Cancer	6.5	6.4	0.004
Dementia	10.4	10.7	0.01

SMD, Standardized mean difference; IQR, Interquartile range.

^*^The length of hospitalization (*n* = 134) and hospitalization cost (*n* = 13,440) were missing in some patients.

^**†**^*p*-value.

In 825,890 patients for whom the NIHSS score was available, neurological severity at presentation showed a substantial imbalance between posterior and anterior circulation ischemic stroke patients. The distribution of NIHSS score strata demonstrated a large SMD indicating markedly higher stroke severity in the anterior circulation group. Posterior circulation ischemic strokes were predominantly mild, with 62.9% presenting with an NIHSS of 0–4, whereas only 34.8% of anterior circulation ischemic strokes fell within this range. In contrast, moderate to severe deficits were significantly more common among anterior circulation ischemic strokes, reflected by higher proportions in the NIHSS 5–15 (37.8% vs. 26.7%) and 15–20 categories (12.4% vs. 3.6%). The highest severity category (NIHSS 21–42) remained uncommon and comparable between groups. Patients with anterior circulation ischemic strokes were markedly more likely to present with aphasia and hemiplegia, whereas posterior circulation ischemic strokes showed higher rates of hemianopsia. Neglect, dysphagia, and stupor also occurred more frequently in anterior circulation ischemic stroke, although with smaller relative differences. In-hospital complications demonstrated generally small SMD indicating broadly comparable rates between groups (see Table 2). Pneumonia, urinary tract infection, deep vein thrombosis, and pulmonary embolism showed minimal imbalance. Modest differences were observed for cerebral hemorrhage, septic shock, respiratory failure, acute kidney injury, and acute myocardial infarction, all slightly more common in anterior circulation stroke. Conversely, tracheostomy and gastrostomy occur at low frequencies in both groups with minimal effect-size differences. Procedural utilization showed some of the largest SMD. Patients with anterior circulation ischemic stroke were substantially more likely to receive intravenous thrombolysis (13.3% vs. 6.1%), undergo cerebral angiography (1.7% vs. 1.3%), or be treated with mechanical thrombectomy (16.4% vs. 4.0%). Carotid revascularization procedures, both stent placement (3.43% vs. 0.24%) and endarterectomy (2.1% vs. 0.2%), were also markedly more frequent among anterior circulation ischemic strokes, whereas vertebrobasilar stent placement was more common in posterior circulation ischemic strokes (0.94% vs. 0.03%). Clinical outcomes demonstrated small to moderate standardized mean differences favoring posterior circulation stroke. Patients with posterior circulation ischemic stroke had lower in-hospital mortality, were more frequently discharged home, and had shorter length of stay and lower hospitalization costs. Use of palliative care was more common in anterior circulation ischemic stroke.

**Table 2:** In-hospital events, procedures, and outcomes.

Variables^*^	Patients with posterior circulation ischemic stroke (*n* = 383,990)	Patients with anterior circulation ischemic stroke (*n* = 1,246,530)	SMD
Clinical deficits			
Aphasia	13.7%	38.7%	0.593
Hemiplegia	32.8%	62.7%	0.627
Neglect	1.9%	6.3%	0.223
Stupor	11.5%	12.6%	0.034
Dysphagia	12.7%	20.0%	0.198
Hemianopsia	9.9%	2.8%	0.294
In-hospital events			
Pneumonia	6.9%	6.8%	0.004
Urinary tract infection	10.9%	11.6%	0.022
Deep vein thrombosis	3.6%	3.7%	0.005
Cerebral hemorrhage	8.0%	11.3%	0.112
Septic shock	7.5%	6.5%	0.039
Cardiac arrest	1.7%	1.3%	0.033
Acute myocardial infarction	6.4%	5.9%	0.021
Pulmonary embolus	1.8%	1.7%	0.008
Respiratory failure	16.7%	17.6%	0.024
Acute kidney injury	19.7%	18.5%	0.031
In-hospital procedures			
Thrombolysis	6.1%	13.3%	0.245
Cerebral angiogram	1.3%	1.7%	0.033
Thrombectomy	4.0%	16.4%	0.419
Vertebrobasilar stent	0.94%	0.03%	0.131
Carotid stent	0.24%	3.43%	0.239
Carotid endarterectomy	0.2%	2.1%	0.179
Intubation	7.8%	8.0%	0.007
Tracheostomy	1.1%	0.9%	0.020
Gastrostomy	0.4%	0.5%	0.015
Outcomes			
Palliative care	9.0%	13.1%	0.131
Discharge home and not palliative	31.2%	26.1%	0.113
In-hospital mortality	7.9%	9.2%	0.047
Median length of stay (IQR)	4 [2 to 8]	5 [3 to 9]	< 0.001^**†**^
Median in-hospital cost (IQR)	$13,680 [8,329 to 26,068]	$17,265 [9,513 to 32,375]	< 0.001^**†**^
NIHSS score strata^**^			
NIHSS score 0–4	62.9%	34.8%	0.586
NIHSS score 5–15	26.7%	37.8%	0.239
NIHSS score 15–20	3.6%	12.4%	0.329
NIHSS score 21–42	6.7%	15.0%	0.269

SMD, Standardized mean difference; IQR, Interquartile range; NIHSS, National Institutes of Health Stroke Scale.

^**†**^*p*-value.

^*^The length of hospitalization (*n* = 134) and hospitalization cost (*n* = 13,440) were missing in some patients.

^**^NIHSS score strata present in 825,890 patients.

Patients with posterior circulation ischemic stroke had higher odds of being discharged home [odds ratio (OR) 1.18, 95% confidence interval (CI) 1.16–1.21, *p* < 0.001, see Table 3] and lower odds of in-hospital mortality/palliative care (OR 0.89, 95% CI: 0.86–0.92, *p* < 0.001). Although statistically significant, the adjusted length of hospitalization for posterior circulation ischemic stroke was shorter than that of anterior circulation ischemic stroke (−0.33, 95% CI: −0.41 to −0.24, *p* < 0.001). Patients with posterior circulation ischemic stroke had a lower cost of hospitalization compared with anterior circulation ischemic stroke, with an adjusted mean decrease of $1,995 (95% CI: 1,684 to 2,305, *p* < 0.001). A contrast in the findings was found in the sensitivity analysis, after adjustment for NIHSS score strata. In-hospital mortality was significantly higher (OR 1.53, 95% CI: 1.45–1.63, *p* = 0.001) in patients with posterior circulation ischemic stroke and significantly lower discharge to home (OR 0.77, 95% CI: 0.75–0.80), *p* < 0.001) as compared to anterior circulation ischemic stroke.

**Table 3:** Adjusted for odds of discharge home without palliative care, in hospital mortality, length of hospitalization, and hospitalization cost in patients with posterior circulation ischemic stroke (NIS 2016–2022).

Outcomes	Patients with anterior circulation ischemic stroke	Patients with posterior circulation ischemic stroke (adjusted for potential confounders)^*^	Patients with posterior circulation ischemic stroke (additionally adjusted for NIHSS score)
Home without palliative care	1.00 (reference)	1.18 (1.16 to 1.21), *p* < 0.001	0.77 (0.75 to 0.80), *p* < 0.001
In-hospital mortality and/or palliative care	1.00 (reference)	0.89 (0.86 to 0.92), *p* < 0.001	1.53 (1.45 to 1.63), *p* < 0.001
Length of stay	0 [reference]	−0.33 [−0.41 to −0.24], *p* < 0.001	0.16 [0.07 to 0.25], *p* = 0.001
Hospitalization cost	0 [reference]	−1,995 [−2,305 to −1,684]	−901 [−1,201 to −601], *p* < 0.001

NIHSS, National Institutes of Health Stroke Scale.

^*^Adjusted for age, sex, race, diabetes mellitus, hypertension, cigarette smoking, hypercholesterolemia, obesity, atrial fibrillation, heart failure, teaching hospital, and rural hospital.

We compared baseline demographic and hospital characteristics, comorbidities, clinical deficits, in-hospital events/procedures, and outcomes between patients in whom NIHSS score were not available (*n* = 804,630) with those in whom they were available (*n* = 825,890) (Supplementary Table 2). Compared with patients in whom NIHSS scores were not available, patients in whom NIHSS scores were available were more frequently admitted at large/teaching hospitals and had higher utilization of thrombolysis and thrombectomy, while patients in whom NIHSS scores were not available had higher complication rates and worse unadjusted outcomes, including higher in-hospital mortality.

There was an increasing temporal trend in the proportion of patients admitted with posterior circulation ischemic stroke from 42,055 in 2016 to 73,755 in 2022 (slope 4,500.357, p trend *p* < 0.001). There was also a temporal trend in the proportion of patients admitted with anterior circulation ischemic stroke, with 144,640 in 2016 and 197,440 in 2022 (slope 8,562.857, p trend *p* < 0.001). There was a trend toward an increase in the proportion of patients with palliative care/in-hospital mortality among patients admitted with posterior circulation ischemic stroke (from 11.3% in 2016 to 13.0% in 2022, p trend *p* < 0.001) and those with anterior circulation ischemic stroke (from 15.5% in 2016 to 17.8% in 2022, p trend *p* < 0.001). There was a temporal trend in the proportion of anterior circulation ischemic stroke patients discharged home (without palliative care) from 25.3% in 2016 to 27.1% in 2022 (p trend *p* < 0.001), but not in patients with posterior circulation ischemic stroke (30.8% in 2016, 32.0% in 2022, p trend *p* < 0.025).

Of the 200 patients in the ICD validation study conducted at the University of Missouri, 148 (74%) had at least one ICD-10 code specifying posterior or anterior circulation ischemic stroke. Using chart review with imaging confirmation as the reference standard, ICD-10 coding for posterior circulation ischemic stroke demonstrated 88.2% sensitivity and 96.5% specificity among those with a localization code. When evaluated across the entire cohort of 200 patients (including those with unspecified localization codes), the sensitivity and specificity for posterior circulation stroke were 65.2% and 97.4%, respectively. For anterior circulation ischemic stroke, ICD-10 coding demonstrated a sensitivity of 96.4% and specificity of 83.3% in the coded subgroup, compared to a sensitivity of 71.4% and specificity of 84.4% in the full cohort.

## Discussion

We found that a total of 383,990 (23.6%) patients were admitted with posterior circulation ischemic stroke over the 7-year period among those in whom location could be identified based on ICD-10 codes. Among patients with posterior circulation ischemic stroke, the in-hospital mortality was 7.9%, with 31.2% of patients discharged home without palliative care. In review the median length of hospitalization for these patients was 4 days, and the median cost of hospitalization was $13,680. Characteristics of patients with posterior circulation ischemic strokes were different from those admitted with anterior circulation ischemic stroke. Patients with posterior circulation ischemic stroke were younger and more likely to be men. Compared with patients with anterior circulation ischemic stroke, patients with posterior circulation ischemic stroke were more likely to have diabetes mellitus and less likely to have concurrent cardiac diseases such as heart failure, myocardial infarction, and atrial fibrillation. Our findings were similar to previous single-center and multicenter registries (7, 22–27). We did not identify any differential relationship with hypertension, which was different from previous studies (7, 24) and may be attributed to the NIS definition of hypertension. The differences in cardiovascular risk factors, particularly, diabetes mellitus, as seen in our study and prior studies (26, 28), support the need for a more individualized approach to secondary prevention in patients with posterior circulation ischemic stroke. For example, pravastatin may be efficacious in those with posterior circulation ischemic stroke based on the Japan Statin Treatment Against Recurrent Stroke (J-STARS) study (6), in which the stroke recurrence rate among patients with posterior circulation ischemic stroke was significantly reduced, but not for those with anterior circulation ischemic stroke suggesting an augmented effect of statin in posterior circulation ischemic stroke patients.

The utilization of IV thrombolysis, cerebral angiogram, thrombectomy, and stent placement or endarterectomy was consistent with previous studies, which have suggested that the use of IV thrombolysis and thrombectomy is lower among patients with posterior circulation ischemic stroke (22, 29–31). This disparity persists despite accumulating evidence supporting the benefit of both IV thrombolysis and thrombectomy in selected patients with posterior circulation ischemic stroke (32–34). The lower utilization of IV thrombolysis in posterior circulation ischemic stroke has been attributed to difficulty in recognizing posterior circulation ischemic stroke symptoms using commonly used prehospital stroke scales and triage systems, and relative insensitivity of CT scans and perfusion imaging in the detection of early ischemia (35–37). This disparity may also be related to the higher rate of admittance to small/non-teaching hospitals observed in posterior circulation stroke patients in our analysis, as it is well known that these smaller hospitals underutilize acute stroke treatments (16, 38, 39). Based on the two large randomized controlled trials demonstrating the therapeutic benefit of thrombectomy in posterior circulation ischemic stroke completed until 2022 (40, 41), it is possible that the proportion of posterior circulation ischemic stroke patients receiving thrombectomy may increase.

We and others have found that patients with posterior circulation ischemic stroke tend to present with less severe deficits as quantified by NIHSS score and a higher proportion of patients in lower NIHSS score strata in our analysis (22, 30, 42–44). The predominance of low NIHSS scores among posterior circulation ischemic stroke patients should not be equated with uniformly mild clinical deficits, because posterior-circulation symptoms (such as gait ataxia, dysarthria, oculomotor deficits) are underweighted in NIHSS, potentially leading to under-triage and delayed access to reperfusion therapies (45). Our results also show a relatively favorable discharge disposition as patients with posterior circulation ischemic stroke are more likely to be discharged home and have lower unadjusted likelihood rates of mortality and/or palliative care admissions compared to those with anterior circulation ischemic stroke. However, we found that the direction of the association between posterior circulation ischemic stroke and outcome measures changed after adjustment for NIHSS score strata (31, 43), and this finding should be interpreted with caution. The NIHSS score is known to incompletely capture neurologic deficits typical of posterior circulation ischemic stroke, as discussed above (45). Consequently, stratification or adjustment by NIHSS score may result in residual confounding or statistical overadjustment. In this context, NIHSS score may function as a mismeasured proxy for stroke severity subject to collider bias or distort the estimated association. As a result, posterior circulation ischemic strokes may appear to have worse outcomes after adjustment, even if their true clinical severity is not fully represented by the NIHSS score. Additionally, these patients were more likely to experience complications such as septic shock, cardiac arrest, acute myocardial infarction, and acute kidney injury, all of which contribute to higher in-hospital mortality (46–48). In contrast to our findings, other comparison studies have either found no difference (22, 30, 49–51) or worse outcomes after adjustment for potential confounders such as NIHSS scores (52, 53).

There are several limitations that need to be considered prior to the interpretation of the results. First, the NIS does not provide data on clinical presentations and findings from neuroimaging tests. Patient identification relies upon the accuracy of ICD-10 coding in the NIS. To address potential misclassification inherent to administrative coding, we performed a local validation study of ICD-10-CM as described above. Overall, these findings suggest that when territory-specific ICD-10 codes are present, they are more reliable for “ruling in” stroke location (high specificity) than for capturing all eligible cases (imperfect sensitivity). Because palliative care was ascertained using ICD-10-CM code Z51.5, under coding or variation in coding practices across hospitals could result in misclassification; however, this approach provides a standardized definition across institutions, and any non-differential misclassification would be expected to bias associations toward the null. In addition, discharge disposition (DISPUNIFORM) reflects disposition at hospital discharge and may not fully capture downstream outcomes after transfer (such as subsequent hospice enrollment or post-acute mortality). However, previous studies have identified a good correlation between discharge disposition to home or other destinations with the modified Rankin scale at 3, 6, and 12 months (54–56). Another limitation in analysis using NIS data is the limited availability of the NIHSS score. The NIHSS score was not available in 804,630 (49.4%) of 1,630,520 patients included in our analysis. Due to differences between patients in whom NIHSS scores were available compared with those in whom NIHSS scores were not available, the findings from NIHSS score strata adjusted analyses should be interpreted as sensitivity analyses with the understanding that they may not be fully generalizable to the entire NIS cohort. In addition, the NIS data does not include the raw NIHSS and instead includes individual ICD-10 codes for score strata. A previous study found excellent agreement between the NIHSS strata ICD-10 codes and the continuous NIHSS score recorded in a stroke registry (57). Finally, we were unable to classify the etiology of ischemic events, such as those related to large vessel atherosclerosis or small vessel disease. The observed increase in the absolute and relative frequency of posterior circulation ischemic stroke between 2016 and 2022 warrants cautious interpretation. Although this pattern could reflect a true epidemiologic shift, alternative explanations are plausible. During this period, MRI utilization and ICD-10 territorial coding specificity increased nationally (58), potentially reducing the proportion of strokes classified as unspecified and improving detection of posterior circulation infarctions. MRI availability may disproportionately increase their ascertainment due to higher MRI sensitivity. Sensitivity analyses accounting for imaging utilization and unspecified territory coding would help clarify this distinction but was not available in the NIS. Clinical presentation variables such as aphasia, hemiplegia, neglect, and other focal deficits were identified using ICD-10 codes and may be incompletely captured, particularly among patients without documented NIHSS score data. This may explain why some neurological deficits were less frequently coded in patients without NIHSS score despite their having higher complication rates and in-hospital mortality. Therefore, findings involving neurological deficits related variables and NIHSS score based sensitivity analyses should be interpreted cautiously. In addition, 65.4% of ischemic stroke admissions lacked territory-specific localization codes and therefore could not be classified as anterior or posterior circulation ischemic stroke. As a result, the reported proportion of posterior circulation ischemic stroke reflects classified cases rather than all acute ischemic stroke admissions. If missing territorial coding differed systematically across hospitals or over time, this may have affected both the estimated proportions of posterior circulation stroke and the observed temporal trends. The proportion of patients without a localizing code in the NIS was higher than in our single center code validation study which may reflect differences in coding practices between university based hospitals and other hospitals.

## Conclusion

Among ischemic stroke admissions with territory-specific localization codes, 23.6% were classified as posterior circulation ischemic strokes. We found that patients with posterior circulation ischemic stroke had differences in discharge home and in-hospital mortality compared with those with anterior circulation ischemic stroke, particularly in the analyses adjusted for NIHSS score strata. Patients with posterior circulation ischemic stroke were substantially less likely to receive intravenous thrombolysis or be treated with thrombectomy. These findings suggest that the clinical burden of posterior circulation ischemic stroke may not be addressed by current triage tools, highlighting a need for infarct location-specific protocols to reduce treatment disparities.

## Data Availability

The original contributions presented in the study are included in the article/Supplementary material, further inquiries can be directed to the corresponding author.
