# Supplementary material for: Proportions, trends, and outcomes of posterior circulation ischemic stroke in the United States
Source: Front Neurol. 2026 May 13;17:1776618. doi: 10.3389/fneur.2026.1776618 (PMC13215130; doi:10.3389/fneur.2026.1776618)
Supplement: Supplementary file 1 [file Table_1.docx]

**Supplementary Figure 1**. Flow diagram of admissions with acute ischemic stroke


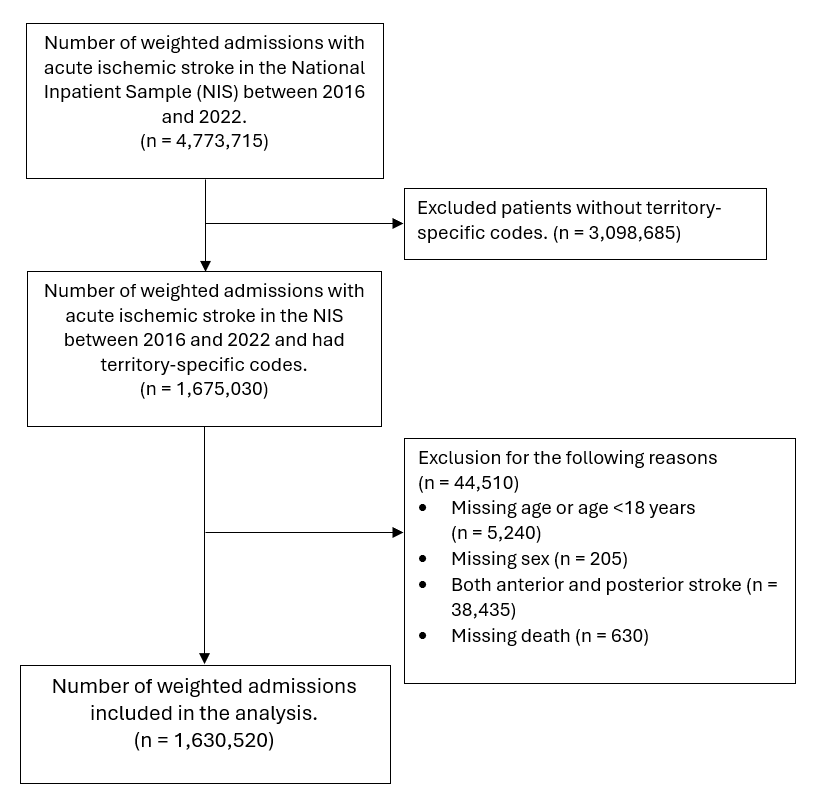


**Supplementary Table 1:** Source of data

| Variables | Variable name or source |
| --- | --- |
| Posterior circulation ischemic stroke | ICD-10 diagnosis codes: I63.432, I63.11, I63.12, I63.213, I63.541, I63.011, I63.012, I63.013, I63.019, I63.01, I63.02, I63.34, I63.542, I63.531, I63.442, I63.549, I63.441, I63.543, I63.431, I63.432, I63.433, I63.532, I63.21, I63.22, I63.533, I63.219, I63.211, I63.443, I63.212, I63.332, and I63.331. |
| Anterior circulation ischemic stroke | ICD-10 diagnosis codes: : I63.512, I63.51, I63.513, I63.519, I63.412, I63.411, I63.312, I63.311, I63.319, I63.419, I63.522, I63.521, I63.529, I63.422, I63.421, I63.429, I63.232, I63.231, I63.321, I63.322, I63.329, I63.131, I63.132, I63.133, I63.139, I63.239, I63.03, I63.031, I63.032, I63.033, and I63.039 |
| Age | AGE |
| Women | FEMALE |
| Race | RACE |
| Hospital bed size | HOSP_BEDSIZE |
| Hospital region | HOSP_REGION |
| Teaching hospital | HOSP_LOCTEACH |
| Rural hospital | PL_NCHS |
| Weekend admission | AWEEKEND |
| Elective | ELECTIVE |
| Transfer | TRAN_IN |
| Primary expected payer | PAY1 |
| ZIP income quartile | ZIPINC_QRTL |
| Cigarette smoking | ICD-10 diagnosis codes: Z720 |
| Alcohol misuse | ICD-10 diagnosis codes: F101 |
| Hypertension | ICD-10 diagnosis codes: I10, I11, I12, I13, I15, I16 |
| Hypercholesterolemia | ICD-10 diagnosis codes: E780, E781, E782, E783, E784, E785 |
| Obesity | ICD-10 diagnosis codes: E660, E661, E662, E668, E669 |
| Diabetes mellitus | ICD-10 diagnosis codes: E08, E09. E10, E11, E13 |
| Previous myocardial infarction | ICD-10 diagnosis codes: I252 |
| Heart failure | ICD-10 diagnosis codes: I0981, I110, I50 |
| Atrial fibrillation | ICD-10 diagnosis codes: I48 |
| Previous stroke | ICD-10 diagnosis codes: I69, Z8673 |
| Peripheral vascular disease | ICD-10 diagnosis codes: I73 |
| Atherosclerosis of the aorta | ICD-10 diagnosis codes: I700 |
| Chronic lung disease | ICD-10 diagnosis codes: J40, J41, J42, J43, J44, J45, J46, J47 |
| Liver failure | ICD-10 diagnosis codes: K72 |
| Chronic kidney disease | ICD-10 diagnosis codes: N18 |
| Cancer | ICD-10 diagnosis codes: C |
| Dementia | ICD-10 diagnosis codes: F01, F02, F03, G30, G31 |
| NIHSS score | ICD-10 diagnosis codes: R2970, R2971, R2972, R2973, R2974 |
| Aphasia | ICD-10 diagnosis codes: I69320, I69920, R4701 |
| Hemiplegia | ICD-10 diagnosis codes: I6935, G81 |
| Neglect | ICD-10 diagnosis codes: R414 |
| Stupor | ICD-10 diagnosis codes: R40 |
| Dysphagia | ICD-10 diagnosis codes: R131 |
| Hemianopsia | ICD-10 diagnosis codes: H5346 |
| Pneumonia | ICD-10 diagnosis codes: J12, J13, J14, J15, J16, J17, J18 |
| Urinary tract infection | ICD-10 diagnosis codes: N300, N309. N341, N342, N390 |
| Deep vein thrombosis | ICD-10 diagnosis codes: I82 |
| Intubation | ICD-10 diagnosis codes: Z9911  ICD-10 procedure codes: 0BH17EZ |
| Tracheostomy | ICD-10 procedure codes: 0B110F4 |
| Gastrostomy | ICD-10 diagnosis codes: Z931 |
| Cerebral hemorrhage | ICD-10 diagnosis codes: I60, |
| Septic shock | ICD-10 diagnosis codes: A41, R6521 |
| Cardiac arrest | ICD-10 diagnosis codes: I46 |
| Systemic inflammatory response syndrome | ICD-10 diagnosis codes: R651 |
| Acute myocardial infarction | ICD-10 diagnosis codes: I21 |
| Pulmonary embolus | ICD-10 diagnosis codes: I26 |
| Respiratory failure | ICD-10 diagnosis codes: J96 |
| Acute kidney injury | ICD-10 diagnosis codes: N17 |
| Thrombolysis | ICD-10 procedure codes: 3E03317 3E04317 3E05317 3E06317 3E08317 3E03017 3E04017 3E05017 3E06017 3E08017 |
| ICD-10 procedure code: Cerebral angiogram | B3181ZZ |
| Thrombectomy | ICD-10 procedure codes: 03CG3ZZ 03CG3Z7 03CG4ZZ 03CH3Z7 03CJ0ZZ 03CJ3ZZ 03CK3Z7 03CK3ZZ 03CL3Z7 03CL3ZZ 03CL0ZZ 03CP3ZZ 03CY3ZZ 00C73ZZ |
| Vertebral artery stent placed | ICD-10 procedure codes: 037Q34Z 037Q35Z 037Q3DZ 037P3ZZ 037P34Z 037P3DZ 03CQ3ZZ 03CP3ZZ |
| Carotid artery stent placed | ICD-10 procedure codes: 037L3 037K3 037J3 037H3 |
| Carotid endarterectomy | ICD-10 procedure codes: 03CL0ZZ 03CJ0ZZ 03CH0ZZ 03CK022 |
| Palliative care | ICD-10 diagnosis codes: Z515 |
| Discharge home and not palliative | No in-hospital death or palliative care |
| In-hospital mortality | DIED |
| Median length of stay | LOS |
| Median in-hospital cost | TOTCH x charge-to-cost ratio |

Abbreviations: ICD, International Classification of Diseases; NIHSS, National Institutes of Health Stroke Scale

**Supplementary Table 2:** Baseline characteristics, in-hospital events, procedures, and outcomes stratified by availability of NIHSS score strata.

| **Variable** | **Patients without NIHSS score strata (n=804,630)** | **Patients with NIHSS score strata (n=825,890)** | **SMD** |
| --- | --- | --- | --- |
| **Demographic characteristics** |  |  |  |
| Age, median (IQR), years | 71 [61 to 81] | 71 [61 to 81] | <0.001† |
| Women | 49.3% | 48.9% | 0.008 |
| **Race** |  |  |  |
| White | 68.4% | 68.9% | 0.011 |
| African American | 16.5% | 16.4% | 0.003 |
| Hispanic | 8.4% | 8.1% | 0.011 |
| Asian or Pacific Islander | 3.2% | 3.2% | 0.000 |
| Native American | 0.5% | 0.5% | 0.000 |
| Other | 2.9% | 2.9% | 0.000 |
| **Hospital characteristics** |  |  |  |
| Small bed size | 15.3% | 12.7% | 0.075 |
| Medium bed size | 27.5% | 24.6% | 0.066 |
| Large bed size | 57.2% | 62.8% | 0.113 |
| Northeast | 18.1% | 17.8% | 0.008 |
| Midwest | 19.1% | 23.4% | 0.106 |
| South | 42.7% | 41.0% | 0.035 |
| West | 20.1% | 17.7% | 0.062 |
| Teaching hospital | 77.5% | 84.5% | 0.178 |
| Rural hospital | 17.1% | 15.9% | 0.032 |
| **Admission Characteristics** |  |  |  |
| Weekend admission | 25.4% | 26.7% | 0.030 |
| Elective admission | 5.9% | 2.6% | 0.162 |
| Transfer admission | 20.4% | 22.9% | 0.061 |
| **Primary Expected Payer, %** |  |  |  |
| Medicare | 64.7% | 63.3% | 0.029 |
| Medicaid | 10.4% | 9.8% | 0.019 |
| Private insurance | 18.5% | 20.0% | 0.039 |
| Self-pay | 3.6% | 4.0% | 0.021 |
| **Cardiovascular risk factors** |  |  |  |
| Cigarette smoking | 1.0% | 0.8% | 0.022 |
| Alcohol misuse | 2.6% | 2.9% | 0.018 |
| Hypertension | 82.2% | 86.0% | 0.106 |
| Hyperlipidemia | 51.3% | 58.3% | 0.141 |
| Obesity | 13.3% | 15.7% | 0.069 |
| Diabetes mellitus | 37.9% | 35.5% | 0.049 |
| Previous myocardial infarction | 7.5% | 7.3% | 0.008 |
| Heart failure | 24.0% | 20.0% | 0.097 |
| Atrial fibrillation | 32.2% | 32.3% | 0.002 |
| Previous stroke | 24.0% | 23.8% | 0.005 |
| Peripheral vascular disease | 4.6% | 3.6% | 0.050 |
| Atherosclerosis of the aorta | 1.8% | 1.7% | 0.008 |
| Chronic lung disease | 17.7% | 15.9% | 0.048 |
| Liver failure | 1.5% | 0.4% | 0.117 |
| Chronic kidney disease | 20.6% | 17.5% | 0.078 |
| Cancer | 7.3% | 5.4% | 0.079 |
| Dementia | 11.5% | 9.8% | 0.055 |
| **Clinical deficits** |  |  |  |
| Aphasia | 27.7% | 37.7% | 0.214 |
| Hemiplegia | 50.7% | 60.5% | 0.198 |
| Neglect | 4.0% | 6.5% | 0.111 |
| Stupor | 8.9% | 15.7% | 0.211 |
| Dysphagia | 17.1% | 19.4% | 0.059 |
| Hemianopsia | 3.5% | 5.5% | 0.096 |
| **In-hospital events** |  |  |  |
| Pneumonia | 9.2% | 4.5% | 0.186 |
| Urinary tract infection | 13.6% | 9.4% | 0.131 |
| Deep vein thrombosis | 4.4% | 2.9% | 0.079 |
| Cerebral hemorrhage | 9.9% | 11.2% | 0.043 |
| Septic shock | 10.1% | 3.5% | 0.261 |
| Cardiac arrest | 1.9% | 0.9% | 0.083 |
| Acute myocardial infarction | 7.4% | 4.7% | 0.113 |
| Pulmonary embolus | 2.2% | 1.4% | 0.060 |
| Respiratory failure | 21.2% | 13.7% | 0.198 |
| Acute kidney injury | 22.4% | 15.2% | 0.184 |
| **In-hospital procedures** |  |  |  |
| Thrombolysis | 7.9% | 15.2% | 0.232 |
| Cerebral angiogram | 1.4% | 1.9% | 0.039 |
| Thrombectomy | 8.9% | 17.9% | 0.267 |
| Vertebrobasilar stent | 0.2% | 0.3% | 0.075 |
| Carotid stent | 2.0% | 3.3% | 0.081 |
| Carotid endarterectomy | 1.8% | 1.4% | 0.032 |
| Intubation | 9.9% | 6.0% | 0.145 |
| Tracheostomy | 1.4% | 0.5% | 0.091 |
| Gastrostomy | 0.6% | 0.3% | 0.045 |
| **Outcomes** |  |  |  |
| Palliative care | 13.6% | 10.8% | 0.086 |
| Discharge home and not palliative | 24.7% | 29.9% | 0.117 |
| In-hospital mortality | 11.1% | 6.8% | 0.151 |
| Median length of stay (IQR) | 5 [3 to 10] | 4 [3 to 8] | <0.001† |
| Median in-hospital cost (IQR) | $15,732 [8,829 to 31,865] | $16,818 [9,521 to 30,440] | <0.001† |

Abbreviations: NIHSS, National Institute of Health Stroke Scale; SMD, Standardized mean difference; IQR, Interquartile range

†p value
